# The Pathophysiology and the Therapeutic Potential of Cannabinoids in Prostate Cancer

**DOI:** 10.3390/cancers13164107

**Published:** 2021-08-15

**Authors:** Kanika Singh, Nazim Nassar, Ava Bachari, Ellen Schanknecht, Srinivasareddy Telukutla, Roby Zomer, Terrence J. Piva, Nitin Mantri

**Affiliations:** 1 The Pangenomics Lab, School of Science, RMIT University, Bundoora, VIC 3083, Australia; s3704285@student.rmit.edu.au (K.S.); s3756626@student.rmit.edu.au (A.B.); s3664278@student.rmit.edu.au (E.S.); srinivasareddy.telukutla@rmit.edu.au (S.T.); 2 School of Health and Biomedical Sciences, RMIT University, Bundoora, VIC 3083, Australia; naz.nassar@rmit.edu.au (N.N.); terry.piva@rmit.edu.au (T.J.P.); 3 MGC Pharmaceuticals Limited, West Perth, WA 6005, Australia; roby@mgcpharma.com.au; 4 The UWA Institute of Agriculture, The University of Western Australia, Perth, WA 6009, Australia

**Keywords:** prostate cancer, cannabis, cannabinoids, cannabinoid receptors, cancer

## Abstract

**Simple Summary:**

Prostate cancer, after lung cancer, is the leading cause of death among men. The incidence rate of prostate cancer varies worldwide between regions and population groups. Its incidence increases with age and is more likely to occur in older men. Although the pathophysiological mechanisms and the etiological factors of prostate cancer development are still poorly understood, there are several factors associated with the risk of developing the disease such as age, family history, lifestyle-related factors (e.g., smoking, diet), and testosterone levels. Cannabinoids are an emerging class of pharmacological molecules that may exert their therapeutic effect against different cancers, including those from the prostate. Several studies have shown that various agonists are able to target cannabinoid receptors exhibited on prostate cancer cells. This affects the release of pro-inflammatory cytokines and has impacts on cell proliferation, cell apoptosis and necroptosis; consequently leading to the development of cancerous cells.

**Abstract:**

Prostate cancer is the second most frequently occurring cancer diagnosed among males. Recent preclinical evidence implicates cannabinoids as powerful regulators of cell growth and differentiation. In this review, we focused on studies that demonstrated anticancer effects of cannabinoids and their possible mechanisms of action in prostate cancer. Besides the palliative effects of cannabinoids, research from the past two decades has demonstrated their promising potential as antitumor agents in a wide variety of cancers. This analysis may provide pharmacological insights into the selection of specific cannabinoids for the development of antitumor drugs for the treatment of prostate cancer.

## 1. Introduction

### 1.1. The Prevalence of Prostate Cancer

The incidence rate of prostate cancer varies worldwide between regions and population groups. According to the global cancer statistics in 2020, 1,414,259 new cases of prostate cancer were registered globally, representing 7.3% of all cancers in men [1]. The age-standardized rate was highest in Oceania (79.1 per 100,000 people) and North America (73.7), followed by Europe (62.1). Conversely, Africa and Asia have incidence rates (26.6 and 11.5, respectively) that are lower than those from developed countries [2]. Prostate cancer incidence increases with age and is more likely to occur in older men. Although only 1 in 350 men under the age of 50 years will be diagnosed with prostate cancer [3], the incidence rate increases to 1 in 52 men between 50 to 59 years. The incidence rate is close to 60% in men over 65 years [4]. Although the pathophysiological mechanisms and the etiological factors of prostate cancer development are still poorly understood, there are several factors associated with the risk of developing the disease such as age, family history, lifestyle-related factors (e.g., smoking, diet), and testosterone levels [5].

### 1.2. Current Treatments in Prostate Cancer

Treatment options for prostate cancer include radical prostatectomy [6], radiation therapy [7], prostate brachytherapy, cryotherapy [8], high intensity focused ultrasound [8], endocrine treatment, laparoscopic prostate surgery, chemotherapy, and androgen-deprivation therapy (ADT) [9]. ADT is utilised as the main treatment for metastatic hormone-sensitive prostate cancer [9]. Cattrini et al. [9], reported that the efficacy of the ADT treatment alone or in combination with conventional drugs such as docetaxel, abiraterone acetate, enzalutamide and apalutamide was low for prostate cancer [9].

**Figure 1:**
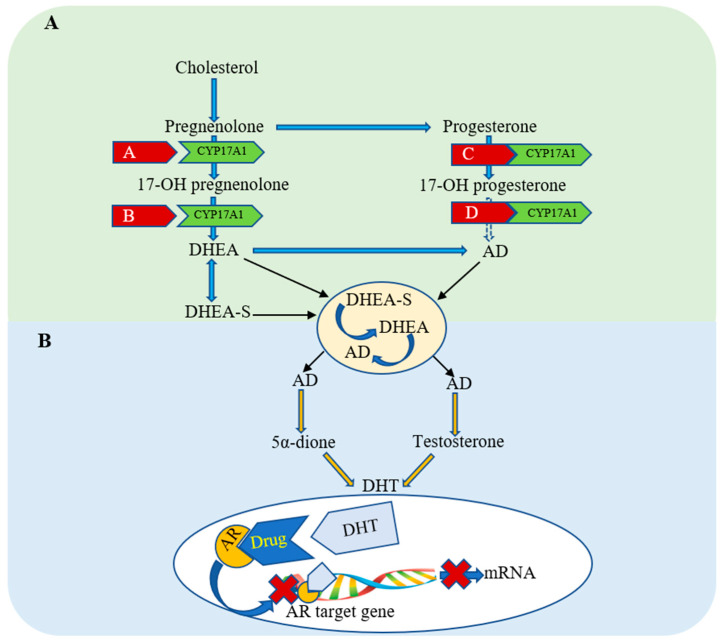
The schematic illustration demonstrates the: (**A**) Pharmacological inhibition of the production process of testostable 17. A1 gene. (**B**) Pharmacological inhibition of androgen receptor (AR) by competing with the prostate converted-adrenal androgens to dihydrotestosterone (DHT).

The correlation between androgen receptors and the production of dihydrotestosterone (DHT) in the prostate is shown in Figure 1. DHT is prevented from binding to the androgen receptor (AR) in the cytoplasm, blocking the triggering of conformational changes and protein–protein interaction that lead to its nuclear translocation. Thus, pharmacological development is focussed on exploring new classes of anti-prostatic cancer drugs that can be utilized solely and/or as adjuvant therapies. The aim is to counteract the emerging resistance to the AR antagonists’ pharmacological activity, lower affinity, greater side effects, and/or expensive approved therapy and to achieve more effective clinical outcomes.

### 1.3. Cannabis sativa and Cannabinoids

Plants have been used as traditional medicines for the treatment of numerous diseases. Recently, bioactive natural products are being used as an alternative therapy for prostate cancer, that offer fewer side effects compared to the conventional treatment of various prostate cancer cell lines such as cannabinoids obtained from the *Cannabis sativa* (*C.* *sativa)* plant. Cannabis has been known by numerous names such as hemp, hashish, bhang, and weed. *C. sativa* is one of the oldest cultivated plants and has been used for thousands of years, not only for its nutritional value but also for medicinal and textile applications [10,11]. It has been used in the treatment of various diseases due to its impacts as an analgesic-hypnotic, antiepileptic and antispasmodic, appetite stimulant, prophylactic, antidepressant, tranquiliser, antiasthmatic, oxytocic (stimulant of uterine contraction during childbirth), topical anesthetic, and antibiotic [12]. Since the 1990s it has emerged as an alternative therapeutic product for treatment of various fatal diseases such as epilepsy, multiple sclerosis, HIV, and cancer.

Cannabinoids are classified into three different categories based on their origin: (a) plant-originated cannabinoids that are found in the marijuana plant such as ∆9-tetrahydrocannabinol (∆^9^-THC) as a psychoactive component of cannabis (phytocannabinoids) and the major non-intoxicating compound cannabidiol (CBD); (b) endocannabinoids including arachidonoyl ethanolamide (anandamide) and 2-arachidonoylglycerol (2-AG), which were found to be products of lipid membrane precursors in humans and animals, forming the endocannabinoid system (ECS) as a critical neuromodulatory system; and (c) synthetic molecules that mimic the structure of either plant or mammalian cannabinoids such as (R)-methanandamide (MET), WIN-55, and JWH-133 [13,14].

The *C. sativa* plant contains more than 500 known plant secondary metabolites, including cannabinoids, terpenes, and flavonoids [15]. The first class consists of cannabinoids such as CBD, Δ^9^-THC, cannabigerol, cannabichromene, cannabicyclol, cannabielsoin, cannabinol, cannabinodiol, and cannabitriol. Cannabinoids are mainly found in the resinous secretion produced by the trichomes of the plant. Over 150 cannabinoids have been isolated to date and derived from cannabigerolic acid [16]. Most of the phytocannabinoids do not possess psychoactive properties [17]. The second class of chemical constituents consists of the nitrogenous compounds such as quaternary bases amides, amines, spermidine alkaloids, amino acids, proteins, glycoproteins, and enzymes, sugars and related compounds (monosaccharides, disaccharides, polysaccharides, cyclitols, aminosugars), hydrocarbons (simple alcohols, simple aldehydes, simple ketones), simple acids (fatty acids, simple esters and lactones, steroids), terpenes (monoterpenes, sesquiterpenes, diterpenes, triterpenes and miscellaneous compounds of terpenoid origin), non-cannabinoid phenols, flavanoidglycosides, vitamins, and pigments. The total number of compounds known to occur in *Cannabis* is 421 with new compounds constantly being discovered and reported [18].

Synthetic cannabinoids (SCs) generate similar psychoactive and physiological effects to that of ∆^9^-THC. Castaneto et al. [19] reported that the pharmacological effects of SCs are up to 100 times more potent than ∆^9^-THC. Furthermore, SCs have great potential for their anti-inflammatory, anti-cancer, analgesic, weight-loss, and anti-seizure effects. Despite this, scientific research into both natural (phytocannabinoids) cannabinoids and SCs has continued. Studies are now being conducted on the potential efficacy of cannabinoids, both natural and synthetic, as anticancer agents and their possible mechanisms of action [15,20,21].

## 2. Cannabinoids and the Entourage Effect

The term “Entourage effect” was first introduced by Mechoulam and his group in 1988 [22]. It refers to the contribution made by compounds (secondary metabolites) in increasing the effectiveness of primary cannabinoids (phyto or endo) in the body. This implies that the total effect of cannabinoids and secondary metabolites is greater than when these compounds are used alone. The whole plant extract has a superior effect in comparison to that of the individual isolated cannabinoids [23,24]. This synergistic interaction may occur between various cannabinoids, known as intra-entourage or between cannabinoids and terpenes, known as inter-entourage [25]. Ben-Shabat et al. reported this phenomenon for the first time in an in vivo study [22]. Their study confirmed the presence of 2-AG endogenous ligands, 2-linoleoyl-glycerol (2-Lino-Gl) and 2-palmitoyl-glycerol (2-Palm-Gl) in various tissues such as the spleen, brain and gut of a mice. They further stated that both the ligands 2-Lino-Gl and 2-Palm-Gl did not bind to the cannabinoid receptors, but they significantly enhanced the binding of 2-AG and its capacity in inhibiting adenylyl cyclase activity. Additionally, these esters considerably changed various motor responses caused by 2-AG in mice such as hypothermia and analgesia [22].

Several studies have further supported this mechanism and demonstrated that all the compounds present in the cannabis plant work together to produce the therapeutic effect [26,27]. Blasco-Benito et al. [27] demonstrated that the botanical drug preparation was more potent than pure ∆^9^-THC in both in vitro and in vivo models of breast cancer. Furthermore, LaVigne et al. [28] reported in a recent in vivo study that a combination of cannabis terpenes (α-humulene and β-pinene) with WIN55,212-2 produced additive effects in the activity of WIN55,212-2 on mice. The findings from these studies suggest that terpenes present in the cannabis plant play a significant role by synergistically improving the activity of isolated cannabinoids. However, so far there are a limited number of studies that have studied this mechanism. More research is warranted to confirm this synergistic activity among various compounds present in the cannabis plant.

## 3. Cannabinoids as Pharmacological Effectors

Cannabinoids are a new emerging class of pharmacological molecules that can exert their therapeutic effect against different tumors including prostatic cancer [13,29,30,31,32]. They bind to specific biological macromolecules exerting different physiological and behavioural responses [14]. Different studies showed that various agonists that target cannabinoid receptors, which are exhibited on cancer cells, can affect the release of anti-inflammatory cytokines, cell proliferation, and cell apoptosis and necroptosis, and consequently the development of cancer cells [29,33,34,35,36].

The pharmacological effect of cannabinoids is mediated through the stimulation of G protein-coupled receptors (GPCRs) called cannabinoid receptor type 1 (CB1) and cannabinoid receptor type 2 (CB2) [37]. CB1 receptors are located in the central nervous system (in the basal ganglia and the limbic system), as well as in non-neural tissues [38,39], while CB2 receptors are the peripheral receptors that are predominantly found in the immune system and the spleen [13,40,41,42]. The pharmacological response depends on the type of endogenous cannabinoid that binds to these receptors. It has been shown that the agonist efficacy of the 2-AG agent is high for CB1, whereas anandamide is a high-efficacy agonist for CB2 receptors [14].

**Figure 2:**
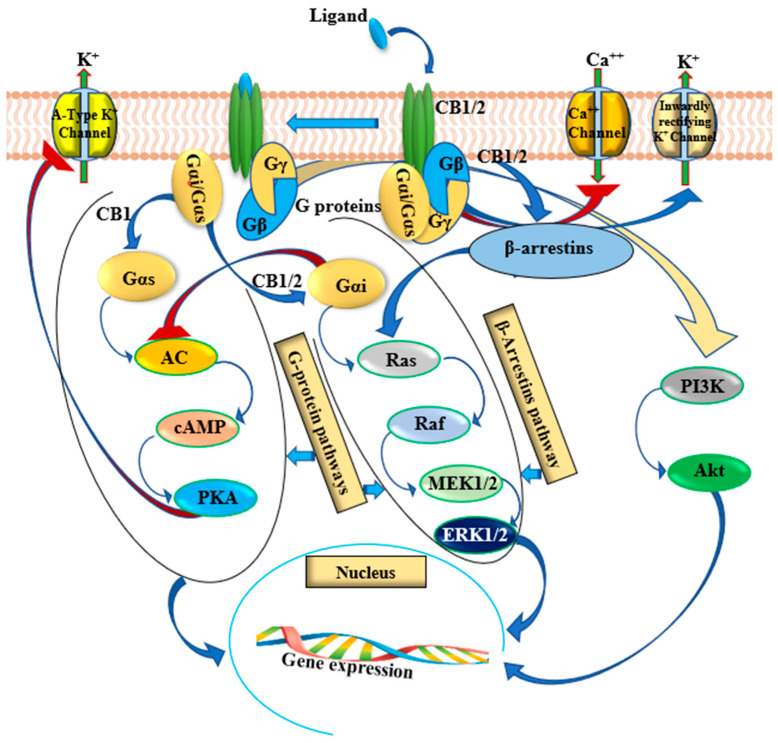
Signaling pathways activated by CB1 and CB2 receptors in prostate cancer cells. The coupling of Gi/o protein heteromers (αβγ subunits) reduce intracellular cyclic adenosine monophosphate (cAMP) levels by inhibiting adenyl cyclase activity. Downregulated protein kinase A (PKA) suppresses PKA mediated signaling events. On dissociation of the α and βγ subunits, phosphoinositol 3-kinase (PI3K) and protein kinase B are stimulated, which further induces the phosphorylation of mitogen activated protein kinase (MAPK). The CB1 receptor can switch G-protein coupling of Gi/o to Gs and Gq and activate several MAPKs such as ERK1/2, p38 and JNK under certain circumstances. CB1 also transduces non-G protein pathway signaling by activating β-arrestins. CB1-mediated signaling is dependent on the ligand and the sub-cellular environment. Arrows indicate stimulation/activation and blunted red arrows indicate inhibition.

Reviewing the literature concerning the pharmacodynamics of a wide range of different cannabinoids that have been researched over recent years, it is far less clear what and how their pharmacological effect is mediated through the convoluted ECS [43]. As discussed earlier, the ECS, including CB1, CB2, and their prominent extended glycoprotein family members form membrane cell-surface receptors, which mediate the pharmacological effect of the different cannabinoids. This distinctive group of glycoproteins consists of diverse G protein-coupled receptors (GPCRs) such as serotonin (5HT) receptors, orphan GPCRs (e.g., GPR18, GPR55, and GPR119), and ligand-gated ion channels [44,45,46]. These GPCRs are combined into GPCR oligomers through intermolecular forces, e.g., covalent bonding between the protomers forming homo/heteromeric dimers/trimers/or tetramers (Figure 2) [44,47,48].

Cannabinoid receptors are well known to form heteromers with a wide range of different receptors [44]. For example, CB1 interacts with GPCRs such as dopamine (D2) receptors, leading to changes in the coupling from Gi to Gs [49]. Moreover, Bonaventura and co-researchers have shown that CB1 can interact simultaneously with adenosine (A2A) and D2 receptors forming A2A/CB1/D2 receptor heteromers that predict differential D2-mediated neurotransmission in the caudate-putamen of the dorsal striatum of the southeastern Asian long-tailed macque monkey *Macaca fascicularis* [50]. Nowadays, various CB1 and CB2-containing heteromeric GPCR oligomers have been found in cancer cells. In breast cancer cells, CB2 was found to form a heteromer with GPR55, and the tyrosine kinase receptor human V-Erb-B2 Avian Erythroblastic Leukemia Viral Oncogene Homolog 2 [44].

Heteromerisation has attracted the attention of scientists in recent years as a prominent potential target in cancer treatment amongst other chronic illnesses. Heteromers exhibit unique physicochemical properties associated with allosteric modulations. Such characteristics support those hybrid molecules to function as viable target sites and strategic platforms for different cannabinoid ligands treating a wide range of chronic diseases, including prostate cancer. Unlike the primary functional forms of plain monomeric receptors, they can lead to various and exemplary pharmacological and cellular functionalities that might influence the antitumor activity of the ECS depending on the combined properties of the complex heteromer and the applied pharmacological agent [51,52,53]. Precisely, the same ligand can display variable affinities to the same receptor (e.g., CB1 or CB2) and subsequently distinctive pharmacological effects, depending on the other partners involved in the unique heteromers in various tissues [53]. That is to say, designing a cannabinoid-based drug to treat prostate cancer is becoming a customised approach based on the heteromeric system, which is more likely to be a “disease-specific” marker [44].

Along with heteromerization as one of the most successfully targeted platforms for selective therapeutic agent innovation, the allostery of ligands is becoming another attractive area to explore cannabinoid-based drug discovery for various medical conditions. Hence, switching from the traditional approach of designing a highly conserved orthostatic agonist/antagonist mimicking the endogenous cannabinoids, e.g., AEA and 2-AG, to flexible allosteric ligands offers a new class of selective therapeutic molecules. These new molecules modulate GPCRs through allosteric sites, resulting in specific heteromeric alterations manifested as a positive/negative allosteric modulator, leading to fine-tuned cellular signaling without undesirable adverse reactions, which has gained tremendous interest during the last decade [54].

### 3.1. Cell Signaling Mediated by Cannabinoid Receptors

As mentioned above, the two cannabinoid receptors possess distinctive molecular structures. They are an integral part of the GPCR system, which undergoes configurational transformation following the binary interactions of the CB1/2 with different agonists, and the association of various intracellular signaling proteins associated with the distinctive complex of the G protein [55,56]. This coupling system allows the CB1 and CB2 receptors to activate multiple pathways, which lead to markedly different responses based on various specific conformational stabilities depending on the cell type [56,57,58,59].

Moreover, cannabinoid receptors can transduce cell signaling through non-G proteins pathways such as β-arrestins, as illustrated in Figure 2. Various GPCR ligands can exert their specific pharmacological effects by activating one specific signaling pathway over another. This phenomenon is called “biased agonism,” manifested by triggering a selective intracellular cascade of events leading to a specific physiological outcome. For further information on this topic please refer to the following references [44,48,52,60].

The activation of cannabinoid receptors CB1 and CB2 results in an inhibition of adenylyl cyclase resulting in reduced cAMP levels in most tissues [61]. Nevertheless, cannabinoid receptors have been shown to control other various signaling pathways that are directly involved in the regulation of cell proliferation and cell death. These signals include the extracellular-signal-regulated-kinase (ERK) as one of the major signaling pathways of the mitogen-activated protein kinase (MAPK) [62], and p38 MAPK [63], phosphatidylinositol 3-kinase (PI3K)/Akt [64], focal adhesion kinase [65], and ceramide and reactive oxygen species (ROS) pathways in prostate cancer [66].

#### 3.1.1. Cannabinoid-Induced Inhibition of Cell Proliferation

The inhibitory effect of cannabinoids on cell proliferation has been known since 1974. Dixit et al. [67] observed a complete halt of spermatogenesis in mice after the daily administration of 2 mg extract of cannabis for 45 days. Long-term exposure to 3–6 mg/kg body weight per day of cannabinoids caused a dose-dependent reduction in gonadotropin releasing hormone 1 receptor protein expression in the pituitary gland, which reduced testosterone levels in male mice [68].

In recent years, attention has been drawn to the pharmacological potential of cannabis on prostate cancer. Several experimental studies have demonstrated and evaluated the effects of various cannabinoids in prostate tissue and prostate cancer cells, which contain both CB1 and CB2 receptors [13,29,30,69,70,71,72]. Louka et al. demonstrated, in DU145 and PC3 prostate cancer cells, that treatment with synthetic cannabinoids, AM-251 and AM-1241, inhibited the proliferation of these cells and increased DNA fragmentation only in DU145 cells [73]. They further stated that in DU145 cells, 48 h treatment with AM-251 and AM-1241 induced caspase dependent apoptosis. However, in PC3 cells, autophagy was induced via the PI3K/Akt/mTOR pathway. Pietrovito et al. demonstrated in a recent in vitro study that treatment with WIN55,212 inhibited migration of cancer associated fibroblasts of prostate cancer and this effect was mediated through the CB2 receptor [70]. Sarfaraz et al. [13] tested the synergistic effect of CB1 and CB2 agonists including WIN-55,212 in vitro on the following cell lines of prostate tissue: CA-human papillomavirus-10, LNCaP, CWR22Rv1, DU145, and PC3. They showed a dose- and time-dependent inhibition of cell growth; moreover, this effect was significantly blocked when the antagonists SR141716 and SR1444528 (CB1 and CB2 receptors, respectively) were introduced [29]. A dose and time-dependent induction of apoptosis, and reduction in PSA levels after the treatment of LNCaP cell line with WIN-55,212 synthetic cannabinoids was observed [29].

Nithipatikom et al. [74] tested the effect of 2-AG and noladin ether on the invasiveness of androgen-independent prostate cancer cell lines including PC3 and DU145 as well as the androgen-dependent cell line LNCaP under the condition of hormone resistance in prostate cancer cells. The results showed that endogenous 2-AG leads to inhibition of androgen-independent PC3 and DU145 cells. This occurred by the invasion of these cells through the activation of the CB1 receptor; nevertheless, inhibition of 2-AG synthesis and antagonising the CB1 receptor results in increased cell invasion [74]. Detailed in vitro and in vivo studies indicating the effects of cannabinoids in prostate cancer are summarized in Table 1.

#### 3.1.2. Cannabinoid-Induced Apoptosis

Administration of cannabinoid receptor agonists such as MET and WIN-55,212 to the culture of LNCaP and PC3 cell lines showed a significant decline of their growth. This reduction in cell viability was associated with inhibition of Akt and activation of ERK [29,37,81]. Furthermore, the treatment of LNCaP cells with CB1 and CB2 agonists such as WIN-55,212 (1–10 µM; 24 h) respectively, showed a significant time- and dose-dependent reduction in cell viability. It also resulted in cell cycle arrest in G0/G1 phase, initiation of p53 and p27/KIP1 genes, down-regulation of cyclins D1, D2, E, and a decrease in the expression of cdk-2, -4, and -6 [37]. It has been suggested that cannabinoids exert their anticancer effects via initiating apoptosis due to the production of ceramide that enhances endoplasmic reticulum (ER) stress [40,59,82]. The accumulation of ceramide triggers the molecular mechanism that is mediated through the mammalian target of rapamycin (mTOR) pathway leading to the autophagy process [83]. Hence, the key mechanism of action by which cannabinoids work in a tumor cell is by inhibiting cancer cell proliferation and induction of cell death by apoptosis [32].

**Figure 3:**
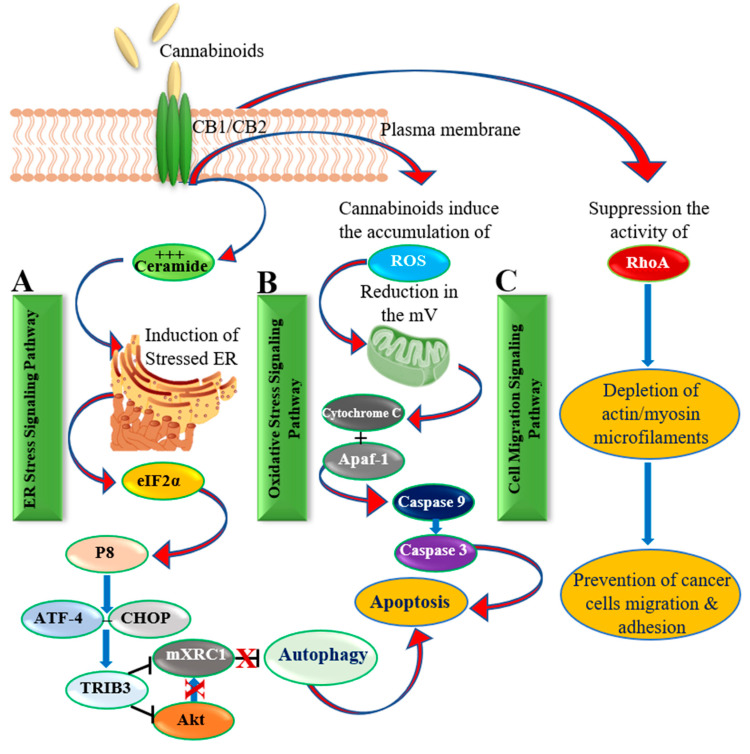
A schematic illustration shows the activation of cannabinoid receptors leading to (**A**). Accumulation of ceramide, which resulted in a build-up in ER stress followed by downstream signaling and induction of apoptosis; (**B**). Increasing the levels of ROS production followed by a reduction in the mitochondrial membrane potential and subsequent release of cytochrome c, and different caspases; (**C**). Inhibition of the RhoA production leading to loss of actin-myosin functionality and consequent reduction in cell migration.

Salazar et al. [83] found that ∆^9^-THC triggered cytosolic ceramide accumulation leading to the activation of ER stress response, resulting in the initiation of factor 2α phosphorylation followed by the autophagy process. Furthermore, they suggested that autophagy resulted from the inhibition of the Akt/mTOR complex 1 (mTORC1) axis, which is mediated via the tribbles homolog 3-dependent signaling (TRIB3) [83] (Figure 3). Olea-Herrero et al. [81] found that the activation of the CB2 receptor by synthetic cannabinoids such as anandamide analogue R(b)-MET, and JWH-015 exerted an antiproliferative effect in PC3 cells as a result of higher ceramide levels. Increased ceramide levels inhibited the Akt-mTOR pathway and activated the initiation factors involved in autophagy regulation as well as the ER stress response. Downregulation of the CB2 receptor led to an increase in viable cells after treatment with anandamide analogues. In vivo treatment with JWH-015 resulted in a significant reduction in tumor growth in mice [81].

Increased ceramide levels followed by an enhancement in ER stress triggered the activation of the caspase cascade leading to apoptosis [69]. Several other studies have demonstrated an increment in caspase-3 activity and reduction of Bcl-2 and Akt levels following the treatment of different prostate cancer cell cultures with endocannabinoid analogues [69,76]. Accumulation of ceramide and the down-regulation of epidermal growth factor receptor (EGFR) have been reported as anti-proliferative and apoptotic effectors generated by the endogenous cannabinoid anandamide in LNCaP, DU145, and PC3 cells [84]. Ceramide has been linked to the upregulation of the stress-regulated protein, which is considered as a pivotal mediator of the anticancer activity of cannabinoids via its induction of apoptosis in those cells. Carracedo et al. [85] have shown that elevated p8 levels are dependent on de novo synthesized ceramide, and that this protein mediates its apoptotic effect through the upregulation of ER stress-related genes ATF-4, TRB3, and CHOP (Figure 3A), which may be potential therapeutic targets to be explored for tumor growth inhibition. Nevertheless, this pathway of apoptotic activation appears limited to tumor cells as normal cells are unaffected due to different cannabinoid-regulatory mechanisms involved in cell death and survival pathways [86].

Apoptosis can be also induced by ROS. Oxidative stress is a phenomenon generated by an imbalance between the overproduction of ROS in the cell and the under-detoxification of these reactive molecules throughout the cell’s antioxidant capacity [87,88]. ROS are immensely reactive molecules that exist in different forms such as anion superoxide (O_2_^−^), hydroxyl radical (OH^.^), and hydrogen peroxide (H_2_O_2_). The high reactivity of these species is due to the presence of unpaired electrons whereby they can generate new reactive molecules or so-called free radicals, interacting with other biological molecules and leading to cellular injury [89,90]. Moderate levels of these free radicals are derived from molecular oxygen throughout the mitochondrial electron transport at the time of the aerobic aspiration. These radicals play a crucial role in cell signaling including triggering apoptosis and gene expression [91]. Nevertheless, different studies have linked oxidative stress with the initiation of a wide range of cancers [69,89,92,93]. The higher ROS levels in prostate cancer cells have been linked with a more invasive and hostile form of these tumors [94]. Targeting ROS production may offer a potential preventative treatment of prostate cancer.

Massi et al. [95] investigated whether the caspase cascade is involved with ROS induction that is mediated by CBD both in vitro and in vivo on U87 glioma cells. They observed that CBD generated a time-dependent caspase-3 apoptotic cell death following the induction of caspases-8 and -9 as well as the release of cytochrome c. They also reported that CBD induced apoptosis and caspase activation occurs via increased ROS levels and glutathione depletion. The effect of the CBD on tumor cells was significantly higher than observed for normal cells due to their ability to generate ROS and activate the caspase cascade in transformed cells as depicted in Figure 3B [95].

The underlying molecular mechanisms of apoptosis that are induced by oxidative stress via cannabinoid receptors have been thoroughly investigated. De Petrocellis et al. [77] tested the effect of cannabinoid extracts on androgenic-receptor cell lines (LNCaP and 22RV1) as well as nonandrogenic-receptor cell lines (DU145 and PC3). They showed significant inhibition in cell viability depending on the type of cannabinoid tested. Cannabinoids at different concentrations (1–10 mM) initiated the intrinsic apoptotic pathway. The pro-apoptotic effect of the experimented cannabinoids on LNCaP cells was only fractional owing to the transient receptor potential melastatin type-8 (TRPM8) antagonism and was chaperoned with the down-regulation of AR, p53 induction, and elevated ROS levels. These findings suggested that cannabinoids trigger the intrinsic apoptotic pathway and cell cycle arrest at the G1-S phase in prostate cancerous cells via ROS as seen in Figure 3B.

Dando et al. [96] used metabolomics to observe the effect of different CB1 and CB2 agonists on pancreatic tumor cells. Elevated AMP-activated protein kinase (AMPK) levels might be due to a ROS-dependent increase of the AMP/ATP ratio [96]. On the other hand, AMPK increases the level of glyceraldehyde 3-phosphate dehydrogenase. Moreover, the results have shown a proportional increase between ROS and NADH, which might be due to an inhibition of the electron respiratory chain and consequently the TCA cycle. Such alterations in cell metabolism, which are initiated by ROS after the mediation of cannabinoids can lead to inhibition of cell growth and the induction of autophagy [96].

#### 3.1.3. Cannabinoid-Induced Inhibition of Cell Motility

Cannabinoids can be potential regulators of cell motility. Manipulation of cannabinoid receptor activity in different cell lines resulted in changes of cell motility [97,98]. It has been shown that manipulation of the phosphorylation of MAPK and focal adhesion kinase intermediates through cannabinoid usage leads to anti-tumoral effects in vitro including cell motility and cell-matrix adhesion in various types of tumors [98,99]. Cannabinoid receptors can activate Rho family proteins leading to an inhibition of cell migration as has been illustrated in Figure 3C.

Nithipatikom et al. [79] investigated the underlying molecular mechanism that inhibited cell migration in PC3, DU145 and LNCaP prostate cancer cells. The CB1 agonist WIN-55,212 significantly reduced RhoA GTPase activity, which was accompanied by the loss of actin/myosin microfilaments and consequent cell migration [79]. Moreover, the activity of the RhoA proteins was significantly increased after the administration of the CB1 antagonist AM251, leading to microfilament formation, which was followed by more cell spreading. Furthermore, the inhibition of RhoA activity due to loss of actin/myosin microfilaments in prostate cancer cell cultures was also observed after the administration of the exogenous CB1 agonist, anandamide [79]. On this basis, reducing cell motility through interference in the mediation of RhoA signaling via cannabinoids receptors represents another pharmacological application against prostate cancer. Furthermore, Roberto et al. reported that the synthetic cannabinoid WIN-55,212 significantly reduced the migration and invasive capacity of PC3 and DU145 prostate cancer cells in a dose dependent manner [71].

## 4. Current Clinical Trials

With respect to cancer, clinical trials demonstrating the effectiveness of cannabinoids are limited. A study in Australia is a phase I/II double blind trial assessing the effect of medicinal cannabis on the quality of life and symptom management in advanced cancer (ACTRN12619001534178) [100]. A randomised phase II trial on the effect of a single dose of CBD on anxiety in breast cancer patients prior to the CT scan to assess tumor burden (NCT04482244) is currently underway [101]. Another phase I/Ib study underway at the University of Kentucky (USA) is determining the safety and effectiveness of Epidiolex (CBD oil) in biochemically recurrent prostate cancer patients [102]. This clinical trial further aims to study whether Epidiolex is a safe treatment for older men with prostate cancer, and if there is any advantage to treating patients with the CBD oil while decreasing PSA and testosterone levels. Finally, the trial aims to identify if Epidiolex could lead to an improvement in the quality of life of prostate cancer patients (NCT04428203) [103]. It would be beneficial to perform further clinical studies using cannabinoids for patients with metastatic prostate cancer. Additionally, not only exploring their effects on prostate cancer but also investigating their analgesic properties for cancer pain associated with metastasis of cancer in the bone would be useful.

## 5. Conclusions

The significant knowledge of anti-tumor and palliative characteristics of cannabinoids acquired by the research community in the past few years has increased the utilization of these molecules as favorable candidates for cancer treatment. Although, the use of cannabis in medicine is restricted due to its psychoactive effects, cannabinoid-based treatments that lack the unwanted side effects are sought after. The lack of knowledge of how cannabis exerts its anticancer effects makes it even more complicated to find what is the best way these compounds can be used without these psychoactive effects. However, there is enough evidence in the literature stating the ability of cannabinoids to induce cell death by various pathways in prostate cancer, but there is still more research needed to be undertaken to understand their mechanism [104,105]. Although cannabinoids may well be able to help with management of prostate cancer, there is still an urgent need to identify the best and the most effective combination for these and other cancers. Furthermore, it is potentially desirable for cannabinoids to work on several hallmarks of cancer at one point in time. Many pre-clinical studies report the anti-tumor activity of cannabinoids [31,72,106]. An important aspect of cannabinoid pharmacology is their selectivity towards cancer cells and not to the normal cells in the body. Another aspect that needs more research is the identification of the mechanism of action by which cannabinoids show synergistic activity/entourage effect with other secondary plant metabolites.

## Figures and Tables

**Table 1:** Effects of cannabinoid treatment on prostate cancer cells in in vitro and in vivo studies.

Cannabinoids	Cannabinoid Receptors	Prostate Cancer Cell Type	Mechanism of Action	Anticancer Effect	In Vitro/In Vivo	Citations
WIN 55-212.2, CBD	CB1, CB2	CAFs, PC3, DU145, LNCaP/PNT-1	Downregulates α-smooth muscle actin and matrix metalloprotease-2 expression, Inhibits CAFs migration	Cannabinoid inhibits CAF migration, impairs the activation and the reactivity of CAFs WIN 55-212.2 ≥ 5 µM and CBD 5 µM induces cell death in prostate cancer cell lines, without affecting healthy prostate epithelial cells	In vitro	[70]
AM-251/ AM-1241		PC3, DU145	Induction of caspase-dependent apoptosis in DU145 cells and autophagy in PC3 cells	Inhibition of the proliferation and reducing viable cell number	In vitro	[73]
WIN55,212-2	CB2	PC3, DU145, LNCaP	Reduction in phosphorylated retinoblastoma (pRb) and Cdk4 expression in a dose-dependent manner; Increase in p27 expression compared to control; WIN55,212-2 exert its anti-proliferative effects partially through the CB2 receptor.	Cannabinoid Induces cell cycle arrest, apoptosis and inhibits proliferation, migration, invasion, and tumor growth in prostate cancer	In vivo/In vitro	[71]
WIN55,212-2	CB2	LNCaP	Downregulated the PI3K/Akt/mTOR signaling pathway; Activation of AMP	Inhibition of neuroendocrine differentiation (NE) and reduction in tumor size	In vitro/In vivo	[75]
AEA, 2-AG, MET	CB1	PC3, Primary tissue samples from patients	Activated caspase-3-Down regulation of Bcl-2- Activated the Erk pathway; Decrease in the activation levels of the Akt pathway; Activation of apoptotic pathway without alteration in cell cycle	Inhibition of cell growth	In vitro	[76]
CBD	LNCaP-TRPM8 PC3–CB1, CB2 DU145–TRPV1 22RV1-TRPV1	LNCaP, 22RV1, DU145, PC3	CBD induces intrinsic apoptotic pathway and upregulated PUMA in all cell lines and AR in LNCaP, 22RV1 Increased expression of p27 and p21, G1/S phase transition in LNCaP, 22RV1, DU145 and PC3 CBD-BDS dose-dependently inhibited the growth of xenografts from LNCaP, but not DU145 cells CBD-BDS dose-dependently inhibited the growth of xenografts from LNCaP but not DU145 cells	Inhibition of cell viability and tumor growth	In vitro/In vivo	[77]
PM49 (synthetic cannabinoid quinone)	PPARγ receptor and partially CB1	LNCaP	ROS production, Cell cycle arrest in G0/G1 phase; Apoptosis induction	Inhibition of cell viability Reduction in tumor growth	In vitro/In vivo	[78]
WIN55212-2	CB1	PC3, DU145	Inhibition of small GTPase RhoA activity and increases the Rac1 and Cdc42 activity; Loss of actin/myosin microfilaments, cell spreading, and cell migration	Decreased cell motility	In vitro	[79]
WIN55212-2, CBD	CB1, CB2	LNCaP	WIN and CBD activate PARP cleavage and induce apoptosis; WIN effects are CB receptor independent; CBD effects are CB1 and CB2 receptor dependent	Cannabinoid induce phosphatases and phosphatase-dependent apoptosis in cancer cell lines. Inhibition of proliferation Inhibition of cell growth	In vitro	[80]
JWH-015, MET	CB2	PC3, DU145, LNCaP	Inhibits Akt-mTOR pathway; Induction of *de novo* synthesis of ceramide and ER stress- proapoptotic effect–Included JNK activation	Inhibition of cell growth Reduction of tumor growth	In vitro/In vivo	[81]
WIN55,212-2	CB1, CB2	LNCaP, PC3	Induction of p53 and p27/KIP1, Down-regulation of cyclins D1, D2, E and E2F1; Decrease in the expression of cdk-2, -4, -6, pRb, DP1 and DP2; Up-regulation of ERK1/2 and inhibition of PI3k/Akt pathways; Increase in Bax/Bcl-2 ratio- Induction of apoptosis; G0/G1 phase cell cycle arrest	Inhibition of cell growth Induction of apoptosis	In vitro	[37]
2AG	CB1	PC3, DU145	Inhibits adenylyl cyclase and decreases activity of PKA; Inhibition of invasion	Inhibition of invasion of prostate cancer cells	In vitro	[74]

Abbreviations: (CAFs): Cancer-associated fibroblasts, (CBD): Cannabidiol, (NE): Neuroendocrine differentiation represents a common feature of prostate cancer, (PrC): primary cell cultures, (MET): Methanandamide, (JNK): (c-Jun N-terminal kinase, (PUMA): p53 upregulated modulator of apoptosis, (BDS): Botanical drug substance, (AEA): Anandamide, (2AG): 2-arachidonoylglycerol, (mTOR): Mammalian target of rapamycin, (ER): Endoplasmic reticulum.

## Data Availability

The data presented in this study are available on request from the corresponding author.

## Abbreviations

A2A	Adenosine
ADT	Androgen-deprivation therapy
AMPK	AMP-activated protein kinase
AR	Androgen receptor
O_2_^−^	Anion superoxide
AMPK	AMP-activated protein kinase
2-AG	2-arachidonoylglycerol
cAMPK	Cyclic adenosine monophosphate
CBD	Cannabidiol
CB1	Cannabinoid receptor type 1
CB2	Cannabinoid receptor type 2
DHEA	Dehydroepiandrosterone
D2	Dopamine
DHT	Dihydrotestosterone
ECS	Endocannabinoid system
ER	Endoplasmic reticulum
ERK	Extracellular-signal-regulated-kinase
EGFR	Epidermal growth factor receptor
GPCRs	G protein-coupled receptors
OH^.^	Hydroxyl radical
H_2_O_2_	Hydrogen peroxide
2-Lino-Gl	2-linoleoyl-glycerol
mTOR	Mammalian target of rapamycin
MET	(R)-methanandamide
MAPK	Mitogen-activated protein kinase
2-Palm-Gl	2-palmitoyl-glycerol
PI3K	Phosphatidylinositol 3-kinase
PKA	Protein kinase A
PKB	Protein kinase B
ROS	Reactive oxygen species
5HT	Serotonin
SC	Synthetic cannabinoids
∆^9^-THC	∆^9^-tetrahydrocannabinol
TRPM8	Transient receptor potential melastatin type-8
TRIB3	Tribbles homolog 3-dependent signaling
